# Automated simultaneous analysis phylogenetics (ASAP): an enabling tool for phlyogenomics

**DOI:** 10.1186/1471-2105-9-103

**Published:** 2008-02-19

**Authors:** Indra Neil Sarkar, Mary G Egan, Gloria Coruzzi, Ernest K Lee, Rob DeSalle

**Affiliations:** 1 MBLWHOI Library, Marine Biological Laboratory, Woods Hole, MA, USA; 2 Department of Biology and Molecular Biology, Montclair State University, Montclair, NJ, USA; 3 Department of Biology, New York University, New York NY, USA; 4 Sackler Institute for Comparative Genomics, American Museum of Natural History, New York NY, USA

## Abstract

**Background:**

The availability of sequences from whole genomes to reconstruct the tree of life has the potential to enable the development of phylogenomic hypotheses in ways that have not been before possible. A significant bottleneck in the analysis of genomic-scale views of the tree of life is the time required for manual curation of genomic data into multi-gene phylogenetic matrices.

**Results:**

To keep pace with the exponentially growing volume of molecular data in the genomic era, we have developed an automated technique, ASAP (Automated Simultaneous Analysis Phylogenetics), to assemble these multigene/multi species matrices and to evaluate the significance of individual genes within the context of a given phylogenetic hypothesis.

**Conclusion:**

Applications of ASAP may enable scientists to re-evaluate species relationships and to develop new phylogenomic hypotheses based on genome-scale data.

## Background

In the post-genomic era, the necessity of developing automated methods for the construction and updating of matrices for complete genome-level phylogenetic analyses of the tree of life have been acknowledged [1]. However, to date a solution for automating gene partition based approaches has been lacking. The main impetus for automated phylogenetic matrix construction is related to the fact that contemporary phylogenetic matrices used to approach the tree of life experience "growing pains" in two dimensions as a result of modern genomics: first, the number of taxa with sequence information "grows" and second the number of data partitions or kinds of genome sequence information "grows" as partial or full genome sequences of species become available.

Several bottlenecks exist in the data acquisition pipeline that can prevent easy construction and updating of phylogenetic matrices. Matrix assembly at the genome scale involves the acquisition (through sequencing or download from databases) of hundreds to thousands of gene regions for the taxa of interest, the formatting of these sequences for use in an alignment program, aligning them, and finally the export of the data partitions into formats used by phylogenetic analysis packages. A phylogenetic analysis package (such as PAUP* [2]) can then be used to infer the phylogenetic tree from the combined matrix. To automate matrix assembly and facilitate the calculation of character-based assessments of tree reliability relative to each of the constituent data partitions, we have developed the Automated Simultaneous Analysis Phylogenetics (ASAP) tool.

While separate analysis of individual gene regions has been used to identify conflicting gene regions, e.g. through the calculation of incongruent length differences [3,4], to suggest their exclusion from a combined analysis, the examination of hidden support in the context of a simultaneous analysis has shown that even these "conflicting" gene regions have hidden support for the simultaneous analysis tree [1,5,6]. ASAP can be used to perform a number of the requisite analyses to calculate hidden support through the generation and execution of PAUP* command. The approach begins by first performing a heuristic tree search for the simultaneous analysis tree. Effective search of tree space for large matrices would preferably be implemented (e.g., using the parsimony ratchet [7]) and there are plans to incorporate such methods into future versions of ASAP. After the generation of the simultaneous tree, ASAP calculates the partitioned branch support (PBS), which identifies the relative contribution of data partitions to branch support within a simultaneous analysis framework. Derived from branch support (BS), in which comparisons are made between the length of the best tree(s) and the shortest trees lacking a given node, PBS is calculated by subtracting the length of the partition on the best tree (*L*_*SA*_; based on the simultaneous analysis of the entire data matrix) from its length on the shortest trees (*L*_*SA*-*N*_; based on simultaneous analysis) lacking a given node:

*PBS *= *L*_*SA*-*N *_- *L*_*SA*_

The difference in lengths is the contribution of a given partition to branch support at that node on the simultaneous analysis tree. PBS can be positive (indicating character support), negative (indicating conflict), or zero (indicating neither support nor conflict). The sum of PBS for all partitions at a node is equal to BS at that node. Next, the hidden support (*HS*) is quantified by ASAP as the difference between the BS for a given node in the simultaneous analysis tree (*BS*_*SA*_) and the sum of the BS values for that node in each of the separately analyzed partitions (*BS*_*P*_):

*HS *= ∑*BS*_*SA *_- ∑*BS*_*P*_

Similarly, partitioned hidden support (*PHBS*) is equal to the PBS (*PBS*_*NP*_; for a given partition at a given node) minus the BS for that node in a separate analysis of that partition (*BS*_*N*_):

*PHBS *= *PBS*_*NP *_- *BS*_*N*_

The sum of the PHBS values across all partitions for a given node is equal to the HBS at that node. The calculation of HS is perhaps the most labour intensive since in each gene region must be analyzed and BS support calculated separately and logged for comparison to the supports calculated for the simultaneous analysis. ASAP saves the results of the calculations into a tab-delimited file, organized by the nodes on the simultaneous analysis tree, which can be imported into popular spreadsheet applications, like Excel. The tab-delimited file contains the support values for each partition at a given node, and can be sorted or filtered from within a spreadsheet application (e.g., one might identify those partitions that contribute the highest support for a given node, versus those that contribute the least).

## Implementation

ASAP is implemented as a series of Perl scripts, and can be installed in most OS X or UNIX environments. This implementation was used to reliably reproduce previously reported gene/partition analyses [8]. Download and installation instructions, along with sample data sets based on previously published gene/partition data sets, are available at the project Website.

ASAP can be incorporated into Websites that serve as repositories for the growing phylogenetic hypotheses generated by the tree of life projects, where specialists can scrutinize the phylogenetic hypotheses and where matrices can be downloaded for further detailed examination.

## Results & Discussion

**Figure 1:**
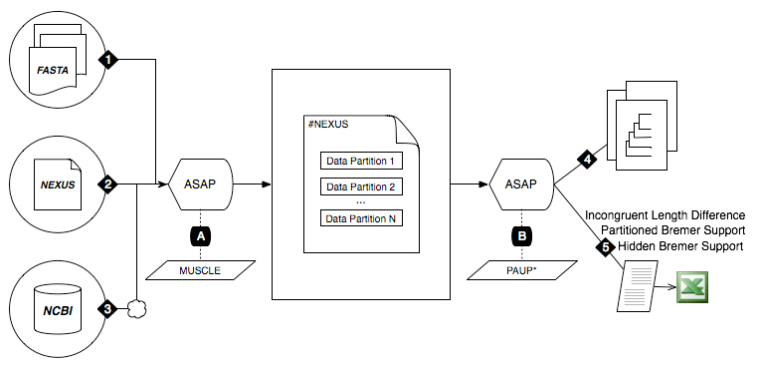
**Overview of ASAP Data Flow**. Data originate in the form of either a collection of systematically annotated FASTA files (1), a NEXUS file with partitions delineated using the CharPars command (2), or a list of NCBI GI/Accession numbers that are organized according to partitions (3). An alignment tool, such as MUSCLE, is applied to unaligned data (A). At this stage, ASAP will assemble a NEXUS file that is a compilation of all the data partitions and performs a tree search to generate a simultaneous analysis tree (4) as well as the requisite tests to determine the hidden branch supports on this tree (5). The results of the hidden branch supports are saved to a tab-delimited text file that can be imported into popular spreadsheet applications like Microsoft Excel.

ASAP takes as input a series of systematically annotated FASTA files (where each file represents a single data partition) or a list of NCBI accession numbers. As graphically depicted in Figure 1, ASAP takes these types of input and systematically generates a NEXUS file with the requisite PAUP* commands to demarcate individual data partitions and accounts for disparities in the data (such as in the cases for missing taxa or missing genes). The resulting NEXUS file is usable by many popular phylogenetic analysis packages, and also organized in a manner to facilitate manual inspection and further curation of the matrix.

ASAP can accommodate both aligned and unaligned sequence data (a subroutine of ASAP will take unaligned sequences and align them using a wide array of sequence alignment tools, by default ASAP is designed to work with MUSCLE [9]). For many researchers, the automation of matrix generation of multiple data types across any number of taxa will facilitate more complete phylogenetic inquiries. As more data are made available from new genome sequencing projects, automated matrix updating can readily be generated using ASAP.

Our approach offers a solution that can be incorporated into pipelines of computer software that automatically scans existing databases, incorporates orthologous sequence information into a "growing" matrix, performs phylogenetic analysis and assesses the affect of the growth of the matrix on the overall phylogenetic hypothesis using a range of data types [10,11]. Importantly, ASAP is not constrained to doing analyses exclusively with molecular data, since a complete phylogenetic analysis may include additional data types such as morphological, behavioral, or ecological characters that are usually coded as discrete character state information.

A number of character-based analyses of the concatenated matrix can be implemented in ASAP, such as partition heterogeneity tests, partitioned Bremer support, bootstrap, jackknife and Bayesian probabilities. These important character-based metrics can be calculated in either a parsimony or likelihood framework. To demonstrate the flexibility of ASAP, we have also incorporated a function to explore how data partition incongruence can be incorporated into the ASAP platform. Other methods of assessing robustness, character support and Bayesian analyses can also be easily interfaced with ASAP and are in preparation as part of the ASAP pipeline. Separate analysis of individual gene regions has been used to identify those regions that conflict with respect to phylogenetic signal (through the calculation of incongruence length differences [3,4] – ILDs). ILD techniques have been extended to identify congruent gene partitions [12]. In contrast to the generally conservative methods for identifying congruent partitions (e.g., via the mILD application [13], incongruence can also be examined through the use of hidden support [6]. ASAP was designed to calculate hidden support through the generation and execution of PAUP* commands.

The approach begins by first performing a heuristic tree search for the tree best supported by the concatenated gene/partition analysis. After a tree is generated from the concatenated gene/partition matrix, ASAP calculates the partitioned branch support (*PBS*), which identifies the relative contribution of each of the data partitions to the overall branch support for the concatenated tree on every branch of that tree. *PBS *can be positive (indicating character support), negative (indicating conflict), or zero (indicating neither support nor conflict). The sum of *PBS *for all partitions at a node is equal to the Branch or Bremer support (*BS*) at that node. The hidden branch support at a node (*HBS*) and the partitioned hidden branch support (*PHBS*) are also quantified by ASAP. Hidden support is the amount of character support that a data set has that is present because of the concatenation of gene partitions. The *PHBS *values at a node are simply the amount of hidden support for a node partitioned to the various genes or genomic regions used in the analyses. The sum of the *PHBS *values across all partitions for a given node is equal to the *HBS *at that node. The calculation of *HBS *is most labor intensive since each gene region must be analyzed and *BS *calculated separately and logged for comparison to the supports calculated for the simultaneous analysis. ASAP saves the results of the calculations into a tab-delimited file, organized by the nodes on the concatenated matrix ('simultaneous') analysis tree, which can be imported into popular spreadsheet applications, like Microsoft Excel. These data can then be used to characterize individual genes and genomic regions for their congruence with the overall evolutionary history of the organisms in the analysis.

As ASAP is character based, the phylogenomic trees constructed using ASAP can be used not only to resolve phylogenetic relationships based on genome scale data, but can also be used to identify the genes and characters associated with the evolution of species and traits. A distance-based approach (e.g. Neighbor Joining or UPGMA) may not allow one to readily use such phylogenomic approaches for gene discovery in the way that can be accomplished with ASAP. These character-based methods allow the researcher to examine the specific gene regions and which individual characters that provide support or conflict with the overall topology of a tree generated from a concatenated matrix. While ASAP is able to assemble large multi-gene matrices for genomic analyses for us in PAUP*, for example, there remain challenges to the analyses of large data sets which would require heuristic rather than exhaustive searches. For this reason, by default, ASAP analysis command files implement rigorous heuristic searches and can accommodate methods such that the parsimony ratchet [8].

At face value, in the case of conflict, this information can be used to identify regions of interest for additional data gathering. The use of these metrics may not be confined to the examination of evolutionary biology hypotheses – an examination of conflict on a simultaneous analysis tree could, for example, identify gene regions whose history is in conflict with phylogenetic history for possibly inferring lateral gene transfer events, or specific characters that could be targeted for loss of function studies.

## Conclusion

The automated simultaneous analysis phylogenetics (ASAP) approach automates the labour and time-intensive task of generating matrices and performing the requisite steps to perform a simultaneous analysis for multi-partition data sets. The current implementation of ASAP (written in Perl) interacts with recent versions of PAUP*, and produces results in a format that can be imported into popular spreadsheet applications like Microsoft Excel for subsequent examination.

## Availability & Requirements

• Project name: *Automated Simultaneous Analysis Phylogenetics (ASAP)*

• Project home page: 

• Operating system(s): *NIX and OS X

• Programming language: *Perl*

• Other requirements: PAUP* (command-line) and MUSCLE

• License: *GNU GPL*

• Any restrictions to use by non-academics: *none*

• Documentation and installation packages (as well as the source code in Perl) for *NIX environments are available at 

## Authors' contributions

INS wrote the scripts in Perl and developed the Website documentation. MGE, RD, and INS developed and evaluated the ASAP algorithms based on existing multi-partition datasets. EKL helped with testing and the ultimate implementation of the ASAP scripts within the context of the OrthologID pipeline. GC provided advice on the implementation of OrthologID within the context of EST data. All authors contributed to the drafting of the manuscript.
